# Integrated 3D-QSAR, molecular docking, and molecular dynamics simulation studies on 1,2,3-triazole based derivatives for designing new acetylcholinesterase inhibitors

**DOI:** 10.3906/kim-2010-34

**Published:** 2021-06-30

**Authors:** Khalil EL KHATABI, Ilham AANOUZ, Reda El-MERNISSI, Atul Kumar SINGH, Mohammed Aziz AJANA, Tahar LAKHLIFI, Shashank KUMAR, Mohammed BOUACHRINE

**Affiliations:** 1 Molecular chemistry and Natural Substances Laboratory, Faculty of Science, University Moulay Ismail, Meknes Morocco; 2 Department of Biochemistry, School of Basic and Applied Sciences, Central University of Punjab, Bathinda India; 3 EST Khenifra, Sultan Moulay Sliman University, Beni Mellal Morocco

**Keywords:** Three-dimensional quantitative structure-activity relationship, molecular docking, molecular dynamics simulations, 1,2,3-triazole, acetylcholinesterase inhibitory activity, Alzheimer’s disease

## Abstract

Alzheimer’s disease (AD) is a multifactorial and polygenic disease. It is the most prevalent reason for dementia in the aging population. A dataset of twenty-six 1,2,3-triazole-based derivatives previously synthetized and evaluated for acetylcholinesterase inhibitory activity were subjected to the three-dimensional quantitative structure-activity relationship (3D-QSAR) study. Good predictability was achieved for comparative molecular field analysis (CoMFA) (Q^2^ = 0.604, R^2^ = 0.863, r_ext_^2^ = 0.701) and comparative molecular similarity indices analysis (CoMSIA) (Q^2^ = 0.606, R^2^ = 0.854, r_ext_^2^ = 0.647). The molecular features characteristics provided by the 3D-QSAR contour plots were quite useful for designing and improving the activity of acetylcholinesterase of this class. Based on these findings, a new series of 1,2,3-triazole based derivatives were designed, among which compound A1 with the highest predictive activity was subjected to detailed molecular docking and compared to the most active compound. The selected compounds were further subjected to 20 ns molecular dynamics (MD) simulations to study the comparative conformation dynamics of the protein after ligand binding, revealing promising results for the designed molecule. Therefore, this study could provide worthy guidance for further experimental analysis of highly effective acetylcholinesterase inhibitors.

## 1. Introduction

Alzheimer’s disease (AD) is a devastating brain disorder characterized by an irreversible loss of memory and damage of cognitive functions. AD typically results in a full deterioration of memory skills and mental activities, leading to the most widespread form of senile dementia and affecting a lot of demented individuals worldwide with increasing tendency [1]. Many etiopathogenic mechanisms including metabolic, genetic, environmental factors, and lifestyle are key pathological features in appearance and progression of the disease [2]. The AD is associated with deficits in cholinergic neurotransmission [3], bio metals dysfunction [4,5], formation of toxic β-amyloid (Aβ) plaques by the deposition of abnormal proteins in the form of these plaques [6], inflammation and increased oxidative stress [7], destabilization of calcium homeostasis [8], and accumulation of tau-protein hyper phosphorylation [9].

Acetylcholine (ACh) is a critical neurotransmitter for specific aspects of brain healthy and cognitive activities. The cholinesterase inhibitors play a vital therapeutic role in elevating ACh levels [10]. That is released at the end of the neuron by the appearance of a nerve impulse, which is transmitted at synapses. Enhancement of the activity of cholinergic neurons seems to be the only way to develop strong medications for reducing disease exacerbation [11]. It is carried out by acetylcholinesterase (AChE) inhibition, the enzyme having control over the hydrolysis acetylcholine [12,13]. The information on crystal structure of AChE is crucial to comprehend its high catalytic effectiveness and atomic basis for the binding of ACh-receptor to recognize ACh [14]. In addition, the clarification of the basic action mechanism of the pharmacological action of these agents would be suitable for further research in the drug design process. The plurality of cholinergic neurotransmission problems is treated by AChE inhibitors, which are the basis of some drugs considered to be the first developed generation drugs to reduce the severity of cognitive disorders [15]. For that reason, the search for new potent acetylcholinesterase inhibitors with improved interactions is highly demanding.

Computational modeling has held upper hand in further research in order to understand the origin and prognosis of neurodegenerative diseases, as every human brain is unique. In silico approaches including 3D-QSAR, molecular docking and MD simulation could offer solutions to important matters which molecular biology alone might not explain. The point of the current research is designing more potent Alzheimer inhibitors with improved binding infinity which could be more effective agents for the management of AD. Therefore, the relationship between structural features of a series of selective 1,2,3-triazole based derivatives identiﬁed as acetylcholinesterase inhibitors and biological activity was revealed by comparative molecular field analysis (CoMFA) and comparative molecular similarity indices analysis (CoMSIA) methods. Consequently, six new compounds with high predicted potency were in silico designed. In addition, molecular docking and molecular dynamics (MD) simulation were performed to ascertain the critical interactions and study the dynamic behavior of ligands in the active sites of AChE related proteins. The integrated approaches have proved to be a promising avenue for drug design, which would provide useful insights into the crucial structural understanding for further synthesis of acetylcholinesterase inhibitors.

## 2. Materials and methods

### 2.1. Data set

**Figure 1:**
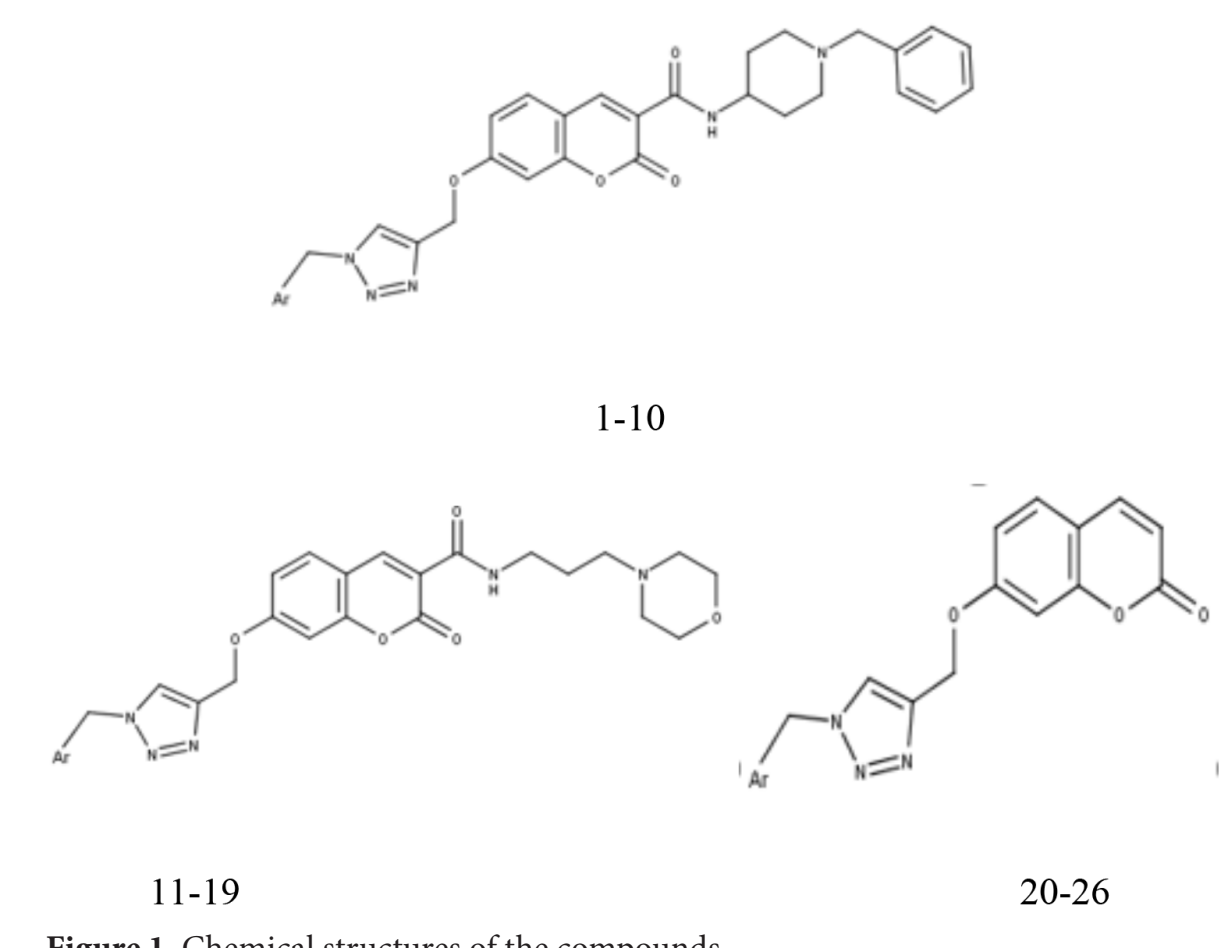
Chemical structures of the compounds.

A series of experimentally reported twenty-six 1,2,3-triazole based derivatives were taken from published studies [16,17] and employed for molecular modeling. The dataset was divided into a training set of 20 molecules (80%) and test set of 6 molecules (20%) to construct and evaluate the models, respectively. The AChE inhibition activities IC50 (µM) were converted into the corresponding pIC50 values, which were further used as dependent variables for quantitative structure-activity relationship (QSAR) analysis. The chemical structures and activity values of the compounds were depicted in Figure 1 and listed in Table 1.

**Table 1:** pIC50 values of the reported 1,2,3-triazole based derivatives against acetylcholinesterase 1-26.

Training/Test	N°	Ar	pIC50
Training	1	H	5.347
Training	2	3,4-Me	5.744
Test	3	4-Me	4.699
Training	4	2-Me	5.431
Training	5	4-OMe	4.437
Training	6	2-Br	5.483
Training	7	2-Cl	5.481
Test	8	2,3-Cl	4.731
Test	9	3,4-Cl	5.690
Training	10	4-F	4.995
Training	11	4-Me	4.361
Training	12	2-Me	5.007
Training	13	2-Br	5.677
Training	14	2,3-Cl	5.298
Training	15	3,4-Cl	3.988
Training	16	4-Cl	4.531
Test	17	3-Cl	4.790
Training	18	4-F	4.789
Test	19	3-F	4.647
Training	20	H	4.432
Training	21	2-F	4.654
Training	22	2-Cl	4.703
Training	23	2-NO2	4.607
Test	24	3-OMe	5.469
Training	25	3-Me	4.301
Training	26	3-F	4.320

### 2.2. Minimization and optimization 

The 3D structure of the studied compounds was sketched using SYBYL-X 2.0 program [18] and minimized under the Tripos standard force field [19], with Gasteiger-Hückel atomic partial charges [20] by the Powell method with 0.01 kcal/mol as the convergence criterion. The compounds were further optimized by density-functional theory (DFT) method B3LYP/6.311 (d, p) basis set level to achieve the equilibrium geometry for each compound [21]. The optimization was performed using Gaussian software (09, Gaussian Inc., Wallingford, CT, USA). 

### 2.3. Molecular alignment

**Figure 2:**
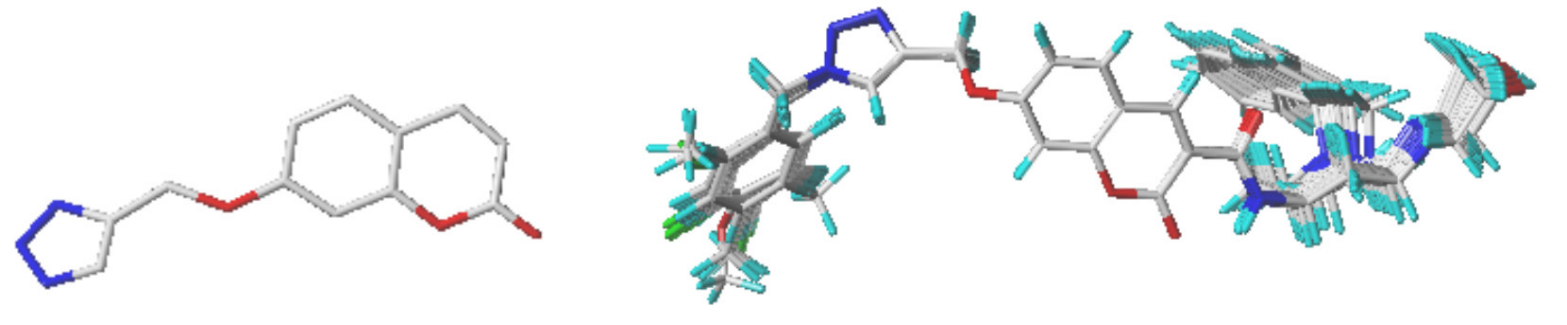
Core and aligned compound using molecule 2 as a template.

The molecular alignment aims to enhance the linearity of 3D-QSAR models. The representative molecule 2 was chosen as template. The remaining molecules were then aligned to common substructure of the template using the simple alignment method in SYBYL [22], which is shown in Figure 2.

### 2.4. 3D QSAR: CoMSIA and CoMFA studies

Based on molecular alignment, 3D-QSAR analysis has been utilized to increase further knowledge of the chemical structures of ligands that correlate well with the bioactivity of the observed interactions [23,24]. These studies were developed to analyze quantitatively, steric, electrostatic, hydrogen bond acceptor (HBA) hydrogen bond donor (HBD), and hydrophobic effects fields. 3D-QSAR analysis was performed by using SYBYL with standard settings. The value of column filtering is set to 2.0 kcal/mol with 30 kcal/mol as the energy cutoff value [25].

### 2.5. Partial least square (PLS) analysis

The partial least squares method [26] is an extension of multiple regression analysis employed to linearly correlate the independent variables (CoMFA and CoMSIA ﬁelds) to dependent variables (pIC50 values). The PLS with leave-one-out cross-validation was carried out using a training set of twenty molecules to obtain simultaneously the cross-validation correlation coefficient (Q^2^) and the optimum number of components (N). Subsequently, the correlation coefﬁcient (R^2^), Fisher test (F) and standard error of estimate (SEE) were obtained by applying noncross validation and without column filtering. In general, (Q^2^ > 0.50 and R^2^ > 0.60) are required conditions for the internal predictability of a QSAR model [27,28]. The best models were also judged based on low optimal number of component and SEE values. To further evaluate the predictability of a model, an external validation was performed using a test set of six molecules, where the required condition (r_ext_^2^ > 0.6) must be satisfied [29].

### 2.6. Y-randomization test

The Y-Randomization test is performed to survey the strength of the generated models [30]. After every iteration, a new QSAR models is created by using the dependent variable (-logIC50) randomly shuffled. The low values of Q^2 ^and R^2 ^of the model indicate that the developed 3D-QSAR model with original data is powerful and is not inferred by chance.

### 2.7. Molecular docking

The molecular docking is a powerful approach, which was conducted by the Surflex-Dock [18] to predict the optimized binding conformation of a ligand and understand the receptor-ligand structural interactions. The obtained results were analyzed using PyMol and Discovery studio 2016 software [31,32]. The ligands and protein preparation steps for the docking protocol were applied to establish molecular docking and predict the binding modes. 

#### 2.7.1. Macromolecule preparation 

The X-ray crystal structure of AChE (PDB code: 1EVE) was downloaded from the RCSB data bank. Its cocrystallized ligand was removed, and the obtained protein structure was utilized for this study. The Discovery Studio 2016 was used to prepare the protein by addition of polar hydrogens and removing water molecules in 1EVE receptor. 

#### 2.7.2. Ligand preparation 

The most active compound of database and the newly designed molecules were docked into the binding pocket of 1EVE receptor. The binding mode between the receptor and docked molecules was studied, compared, and selected for further analysis.

### 2.8. Molecular dynamics (MD) simulation

The AChE complexed with drug candidates were subjected to MD simulation to understand the dynamic behaviors and conformational changes of ligands. GROMACS 5.1.1 software package with gromos54a7 force field was used to run 20 nanoseconds MD simulation on docked complexes of inhibitor and protein. Ligand parameters were generated with the same force field using PRODRG server [33]. The cubic simulation box was generated by gmx editconf tool. The system was solvated with SPC water model using gmx solvate tool. The electro-neutralization of the system was performed using gmx genion tool. Afterwards, energy minimization was carried out to eliminate steric clashes and optimize of structure. Following energy minimization, equilibration was performed in two steps. The NVT equilibration was performed for 100 picoseconds at 300 K to stabilize the temperature of the system. This was followed by 100 picoseconds NPT equilibration at 1 bar pressure. The pressure coupling at 1 bar was maintained using Parrinello–Rahman barostat [34]. Using the LINear Constraint Solver (LINCS) algorithm, bond lengths were kept conserved [35]. Particle mesh ewald (PME) was applied to deal with long-distance electrostatics interactions [36]. Finally, the 20 ns MD simulation was applied for each complex with desired temperature and pressure on a Linux machine with Intel core i-7 processor (32 GB RAM).

## 3. Results and discussion 

### 3.1. Statistical analysis and validation

Based on the 3D-QSAR modeling, it seemed that the created models have a credible fitting for predicting new anti-acetylcholinesterase agents as contained in the PLS analyses summary (Table 2). The experimental and predicted pIC50 are shown in Table 3. Obviously, highly predictive abilities were assessed for both models. In the CoMFA model, the resulting crossvalidated coefficient Q^2^ is 0.604 with 4 as principal components, N. The R^2^, SEE and F-test values of the best CoMFA model were 0.863, 0.16 and 93.62, respectively, while the proportions of steric and electrostatic contributions in the model are, respectively, at 67.9% and 32.1%. The optimal CoMSIA model generated an Q^2^ of 0.606 with 4 as principal components, R^2^ of 0.854, a low SEE of 0.17 and 99.96 as F value. The proportions of steric, electrostatic, HBA, HBD, and hydrophobic contributions accounted for 5.3%, 21.4%, 14.7%, 33.5%, and 25%, respectively. Moreover, highly external prediction ability was achieved, in which the external validation coefficient r_ext_^2^ value for CoMFA was 0.701 and 0.647 belonged to CoMSIA. The high Q^2^, R^2^ and r_ext_^2 ^values along with low SEE value and an optimum number of components, suggest that both models possess high predictive abilities and signiﬁcant statistical reliability of the QSAR models. 

**Table 2:** PLS statistics parameters.

Model	Q2	R2	SEE	F	N	rext2	Fractions				
							Ster	Elect	Acc	Don	Hyd
CoMFA	0.604	0.863	0.16	93.62	4	0.701	0.679	0.321	-	-	-
CoMSIA	0.606	0.854	0.17	99.96	4	0.647	0.053	0.214	0.147	0.335	0.250

**Table 3:** Experimental and predicted pIC50 of twenty-six 1,2,3-triazole based derivatives.

Training/Test	N°	pIC50	CoMFA	CoMSIA
			predicted	predicted
Training	1	5.347	5.462	5.458
Training	2	5.744	5.673	5.664
Test	3	4.699	5.037	5.031
Training	4	5.431	5.507	5.522
Training	5	4.437	4.373	4.361
Training	6	5.483	5.534	5.529
Training	7	5.481	5.528	5.522
Test	8	4.731	5.209	5.200
Test	9	5.690	5.258	5.264
Training	10	4.995	4.904	4.905
Training	11	4.361	4.465	4.462
Training	12	5.007	5.069	5.084
Training	13	5.677	5.785	5.779
Training	14	5.298	5.197	5.178
Training	15	3.988	4.237	4.239
Training	16	4.531	4.334	4.331
Test	17	4.790	4.710	4.706
Training	18	4.789	5.673	4.793
Test	19	4.647	4.682	4.687
Training	20	4.432	4.409	4.408
Training	21	4.654	4.604	4.611
Training	22	4.703	4.713	4.721
Training	23	4.607	4.601	4.610
Test	24	5.469	5.448	5.459
Training	25	4.301	4.311	4.308
Training	26	4.320	4.307	4.310

### 3.2. Contour map analysis

**Figure 3:**
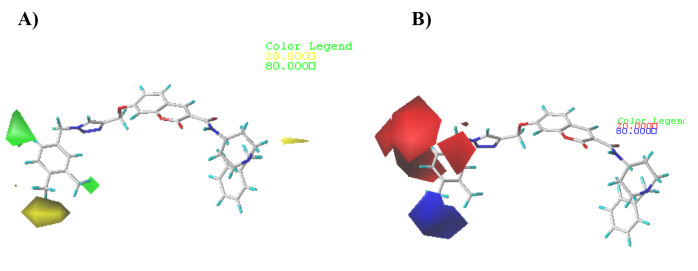
Contour maps of CoMFA analysis for compound 2. A) Steric fields; B) Electrostatic fields.

**Figure 4:**
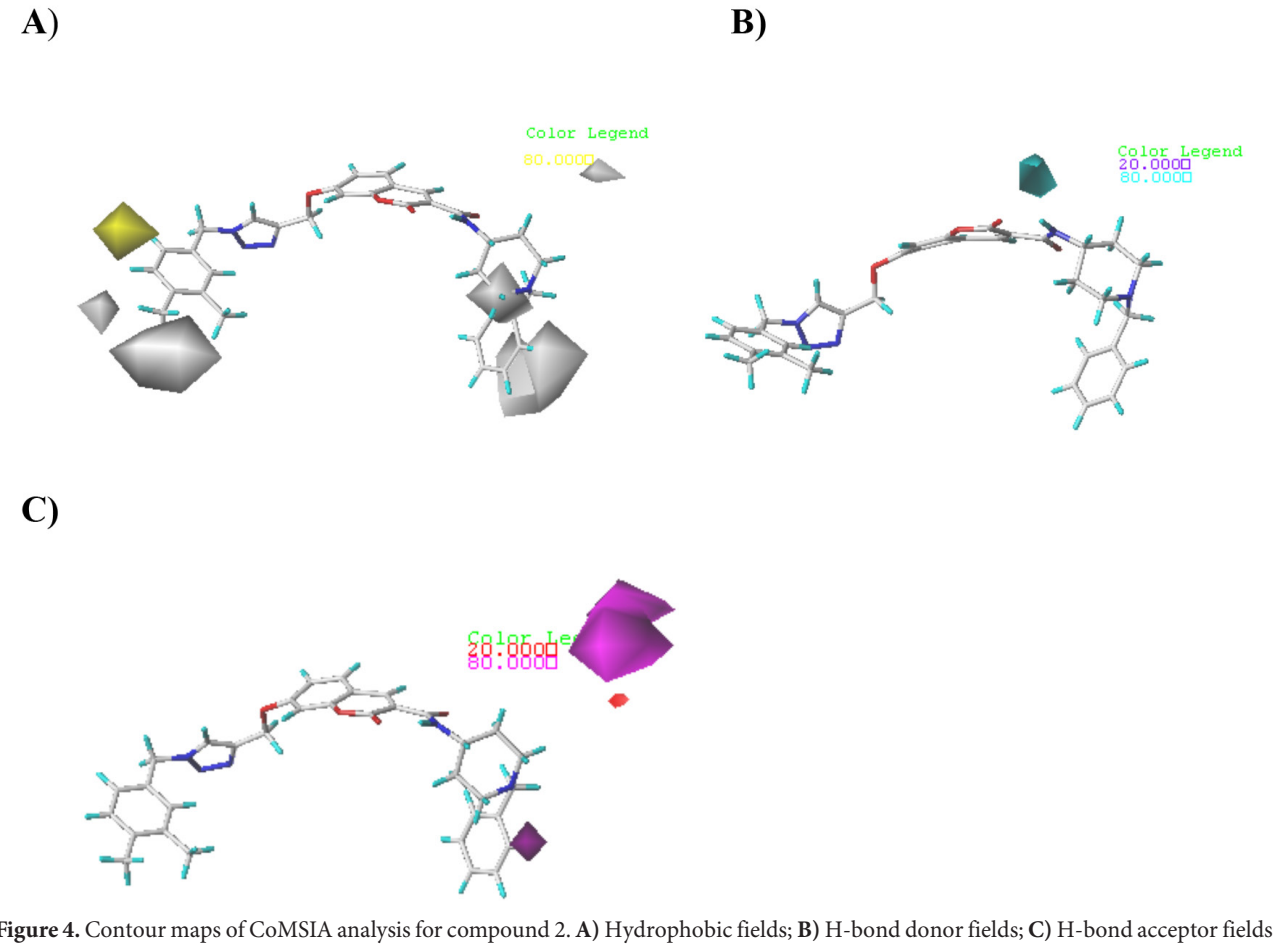
Contour maps of CoMSIA analysis for compound 2. A) Hydrophobic fields; B) H-bond donor fields; C) H-bond acceptor fields

To identify the structure requirements contributing to the binding affinity, the CoMFA/CoMSIA contour maps were generated, which could provide an increase in the biological activity of the molecules. The modification in certain area according to the useful information provided by the contour maps would rationally guide lead optimization. Compound 2 was used as a reference structure since it is the most active compound and then superposed over the CoMFA and CoMSIA contour maps as displayed in Figures 3 and 4, respectively.

#### 3.2.1. CoMFA contour maps

CoMFA steric interactions are displayed in green (80% contribution) and yellow (20% contribution) colored contours. The bulky substituents are favored around green regions, while yellow regions’ bulky groups are unfavored. As depicted in Figure 3-A, two small green contour maps located at C-3 and C-6 positions of the phenyl, indicate sterically favorable bulky substituents at these positions, which could increase activity. A yellow contour map is covered the C-4 position of the phenyl ring, indicating that position is not favorable for larger substituent, which could be demonstrated by compound **5** . For example, the compound **9** (pIC50 = 5.690) with OCH_3_ group at the C-4 position of the phenyl ring showed lowest activity than those with no or less bulky substitute at the same position.

CoMFA electrostatic interactions are displayed in red (20% contribution) and blue (80% contribution) colored contours. The electropositive charge groups are favorable in the blue regions of electrostatic contours for enhancing the inhibitory activity, while the red contour may lead to an increase in inhibitory activity of the electronegative charges as shown in (Figure 3-B). Two red contour maps are seen near C-1, C-2, and C-6 positions of the phenyl ring, suggested that electronegative groups at these positions would exhibit good anti-acetylcholinesterase activity, this can explain why the activity of compounds **6** and **7** with an electronegative substituent in C-2 position of phenyl ring, showed a slight increase of inhibition affinity. On the other hand, a medium sized blue contour is observed close to the C-3 and C-4 positions, which suggested that electropositive groups at these positions are favorable to increase the inhibitory potency of 1,2,3-triazole based analogs.

#### 3.2.2. CoMSIA contour map 

The CoMSIA contour plot analysis was carried out to describe the important molecular properties of the steric, electrostatic, H-bonding, and hydrophobic interaction fields. Since the steric and electrostatic contour maps obtained from the CoMFA and CoMSIA model were consistent, only the hydrophobic and H-bonding contour maps were described and analyzed here.

As mapped in Figure 4-A, the yellow areas (80% contribution) are where hydrophobic groups predicted to enhance biological activity, whereas white domains (20% contribution) represent areas where hydrophobic groups are predicted to be detrimental to activity. A yellow contour can be observed around the C-6 position of the phenyl ring, which indicates that replacing this position with hydrophobic groups may increase the activity. In contrast, a white contour near the C3 and C4 positions revealed hydrophobic groups at these positions would result in the loss of the bioactivity. For example, derivatives **17** , **19** , **25,** and **26** (pIC50 = 4.301–4.790) bearing hydrophobic groups (i.e., F, Cl, and Me) at C-3 position presented distinct decrease. Moreover, two medium sized white contours are observed on the other side of the molecule close to benzylpiperidine which suggested that hydrophilic groups at these positions are favorable to increase the inhibitory potency. 

The cyan (80% contribution) and purple (20% contribution) contours (Figure 4-B) represented the desired and undesired positions for donating hydrogen bond, respectively. A cyan contour is seen on the bridge between the piperidine and benzylpiperidine indicates that hydrogen bond donors at this area could improve the potency. 

The favorable and unfavorable positions for HBA groups are shown as magenta (80% contribution) and red (20% contribution) contours, respectively (Figure 4-C). The magenta contour around benzylpiperidine illustrated favorable region where HBA groups are beneficial for the biological activity, while a very small red contour located at piperidine implies that the HBA groups at this position would decrease the inhibitory activity. 

### 3.3. Y-randomization test

Table 4 shows the results of nine random shuffles for the Y-randomization test. The Q^2^ and R^2^ obtained by the nine iteration were ranging from –0.014 to 0.227 and 0.116 to 0.402, respectively, for CoMFA, while for CoMSIA, the Q^2^ and R^2^ were in the range (from –0.099 to 0.235) and (0.137–0.329), respectively. This indicates that the developed models are robust and are not inferred by chance correlations.

**Table 4:** Q2 and R2 values of the Y-randomization test.

Iteration	CoMFA		CoMSIA	
	Q2	R2	Q2	R2
1	0.182	0.288	0.148	0.294
2	0.025	0.187	0.021	0.192
3	0.109	0.322	0.159	0.329
4	0.121	0.244	0.103	0.288
5	–0.014	0.158	–0.099	0.166
6	0.098	0.116	0.102	0.137
7	0.227	0.402	0.199	0.296
8	0.201	0.275	0.235	0.301
9	0.091	0.128	0.102	0.141

### 3.4. SAR summarized results

**Figure 5:**
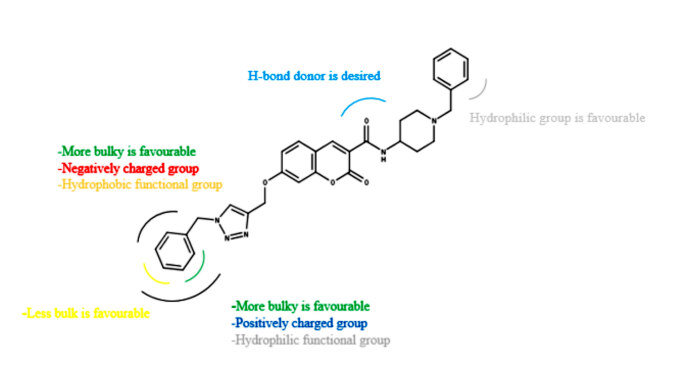
SAR summarized results from the 3D-QSAR study

Figure 5 summarized the information provided by CoMFA and CoMSIA studies, which could supply some meaningful clues to design new molecules with high predictive activity. Various modifications were tried on certain areas by use of outcome of the SAR results.

### 3.5. Newly designed compounds 

**Figure 6:**
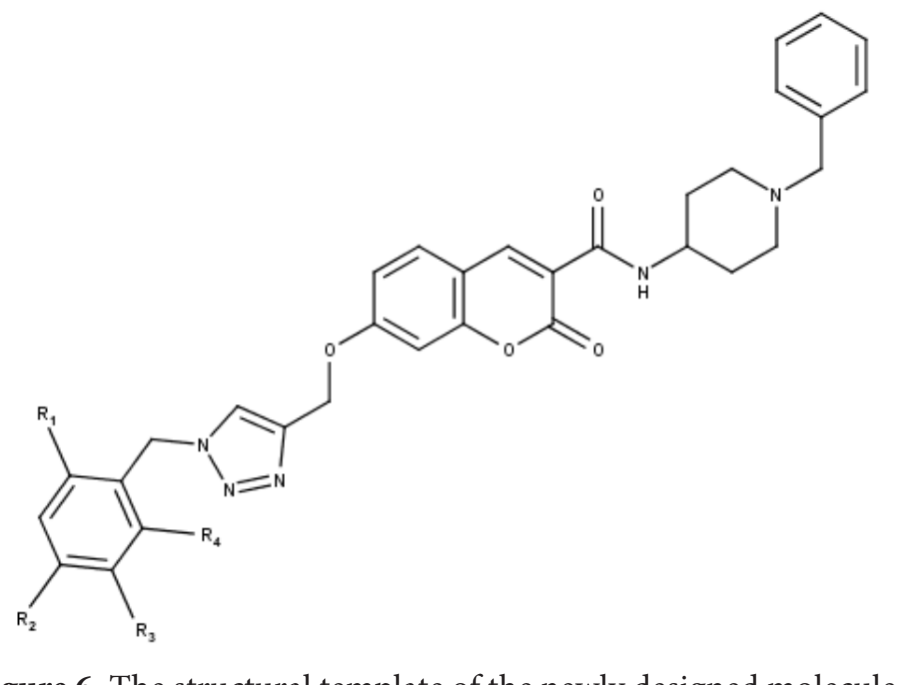
The structural template of the newly designed molecules.

According to the main structure–activity relationship revealed by the present study, six (A1-A6) new 1,2,3-triazole based derivatives were in silico designed by modifying chemical structure of compound 2. Figure 6 shows the structural template of the newly designed molecules. The predicted activity values of the newly designed compounds were in the range (6.026–6.569) and (6.062–6.597) for CoMFA and CoMSIA models, respectively. All the designed molecules showed greater predicted activities not only than that of compound 2, but also than its experimental activity (pIC50 = 5.744) as shown in Table 5. 

**Table 5:** Predicted pIC50 values of the newly designed molecules.

N°	R1	R2	R3	R4	Predicted pIC50		Total score
					CoMFA	CoMSIA	
The most active compound							
Comp.2	H	CH3	CH3	H	5.673	5.664	5.8942
The newly designed compounds							
A1	NO2	H	NH2	H	6.569	6.597	6.7088
A2	NO2	OH	NH2	H	6.470	6.495	6.6201
A3	NO2	H	OH	H	6.442	6.468	6.6194
A4	NO2	H	NH2	OH	6.407	6.467	6.6188
A5	NO2	H	H	OH	6.204	6.239	6.4112
A6	NO2	H	H	H	6.026	6.062	6.3082

### 3.6. Docking results

**Figure 7:**
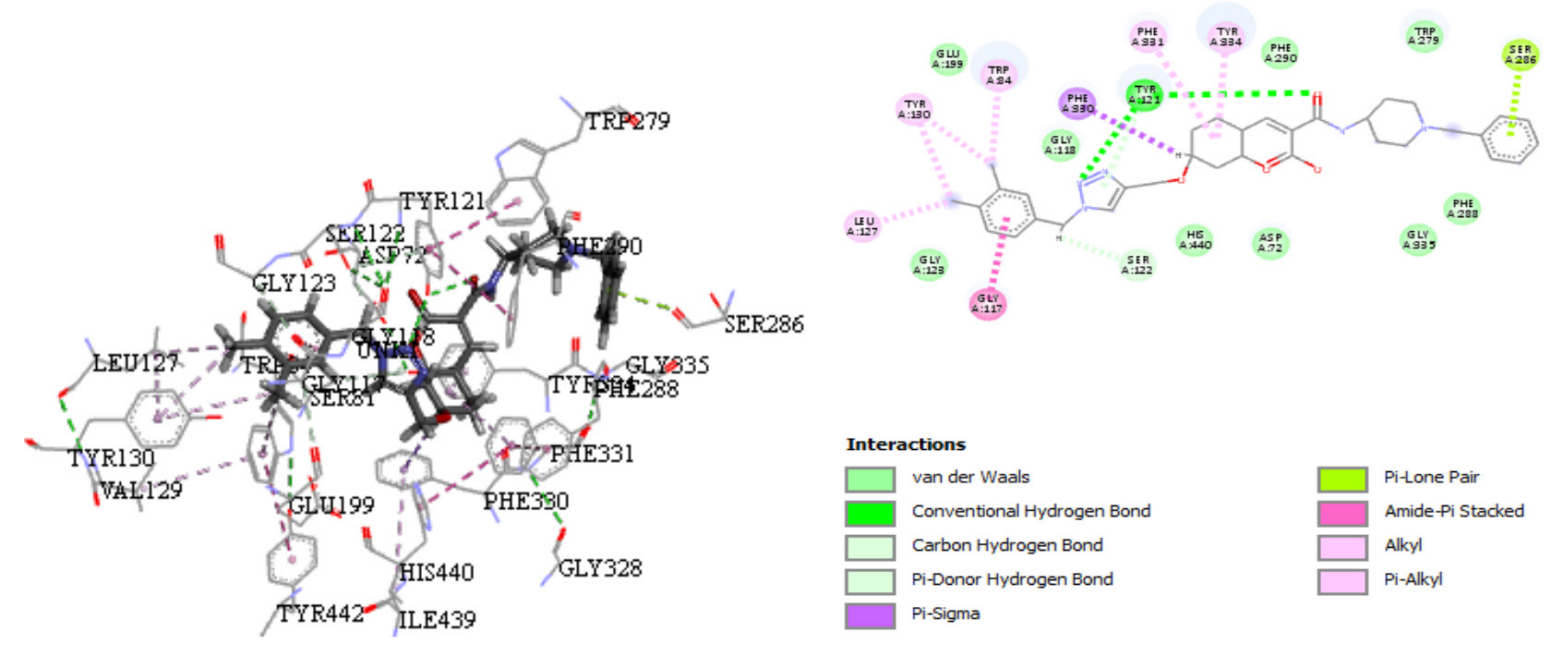
Docking interactions of the active compound 2 with the receptor

The most active compound and designed compounds were molecularly docked with the X-ray crystallized complex of AChE (PDB:1EVE) using Surﬂex-dock to gain deeper insight into the binding modes and explore the interactions between the protein and these inhibitors. Compound **2** and the proposed compound **A1** were further subjected to detailed molecular analysis and compared. They were all well-superimposed in the ligand-binding pocket of the protein. The proposed compound **A1** was scored as high as 6.7088. It was found higher than that of compound 2 (total score = 5.8942). As shown in the two-dimensional diagram of the molecular docking (Figure 7), the 1,2,3-triazole linker and the C-O of amide bond made both a hydrogen bond with TYR120. Moreover, the NH on the bridge between 1,2,3-triazole linker and the phenyl substituted by two methyl was in Pi-donor hydrogen bond contact with SER122. On the other side of the ligand, benzyl moiety made a Pi Lone Pair interaction with SER286.

**Figure 8:**
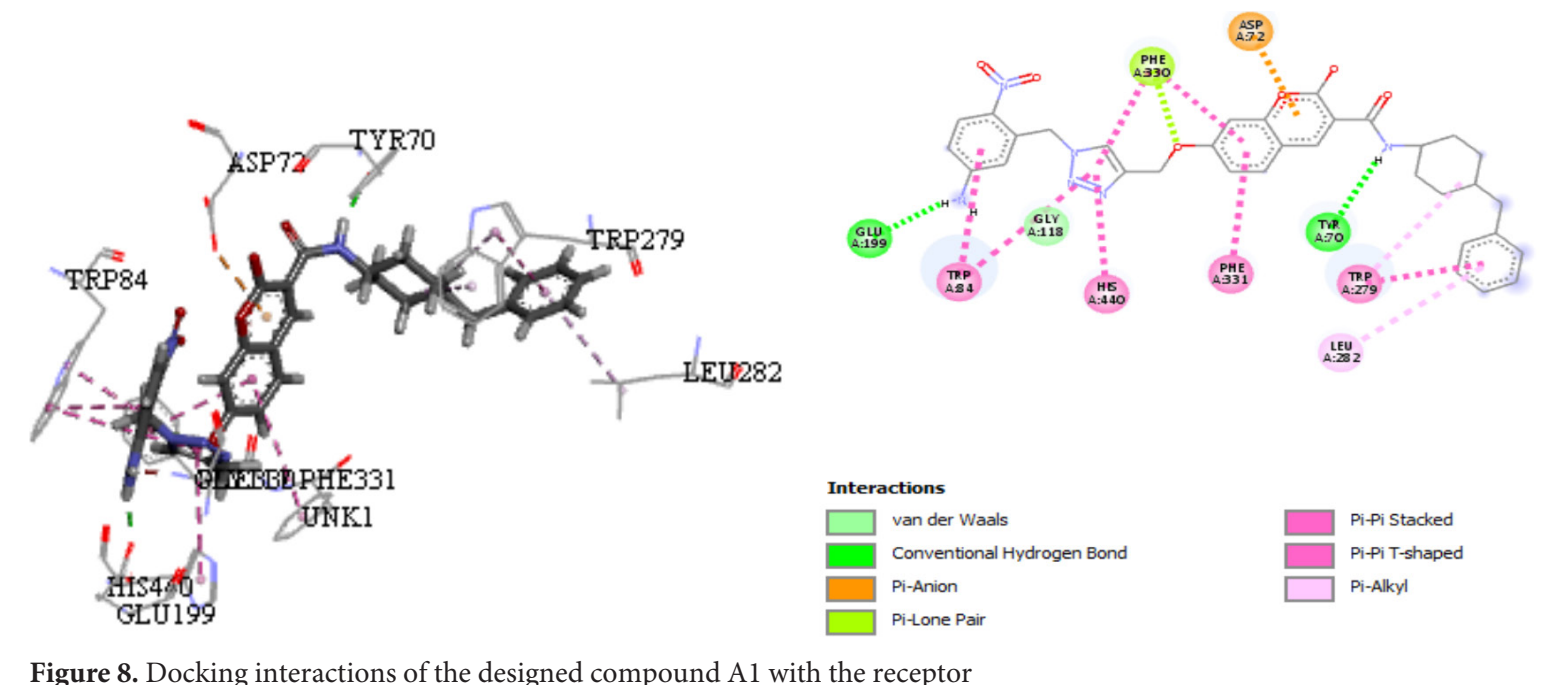
Docking interactions of the designed compound A1 with the receptor

The results of docking showed favorable and convenient interactions for the proposed compound **A1** (Figure 8) by presenting a conventional hydrogen bond. The NH of amide bond made a hydrogen bond with TYR70; the NH substituent in C4 position of the benzyl ring connected to 1,2,3-triazole formed a hydrogen bond interaction with GLU199. Moreover, the coumarin fragment with planar structure bound to the aromatic amino acid PHE331 by a π-π stacking interaction.

Furthermore, the docking results had been compared with the QSAR results to confirm mutually the correlation. The binding interactions match well with the results of electrostatic and hydrophobic contour maps. These findings support the selected pose of the proposed compound **A1,** which would form a stronger inhibitory effect on the receptor protein. 

### 3.7. Molecular dynamics simulation

We utilized the X-ray crystal structure of AChE protein (PDB ID: 1EVE) to dock the 1EVE compounds. These virtual structures were subjected to 20 ns MD simulations to study the comparative conformation dynamics of the protein after ligand binding. Energy, temperature, pressure, and density of the compound bound protein were stable during the 20 ns simulation. Table 6 shows the average values of various MD related properties.

**Table 6:** Average values of various MD related properties.

S. No.	Property	Protein-active molecule complex	Protein designed molecule complex
1	RMSD (Backbone)	0.2565 ± 0.0341 nm	0.2540 ± 0.0280 nm
2	RMSD (Protein)	0.3129 ± 0.0336 nm	0.3090 ± 0.0308 nm
3	RMSF	0.1531 ± 0.0731 nm	0.1431 ± 0.0694 nm
4	Radius of gyration	2.3604 ± 0.0081 nm	2.3600 ± 0.0072 nm
5	SASA	235.5676 ± 4.5732 nm2	241.7229 ± 3.5879 nm2
6	Hydrogen bonds (Protein-protein)	399.9980 ± 10.4525	397.3213 ± 9.7869

**Figure 9:**
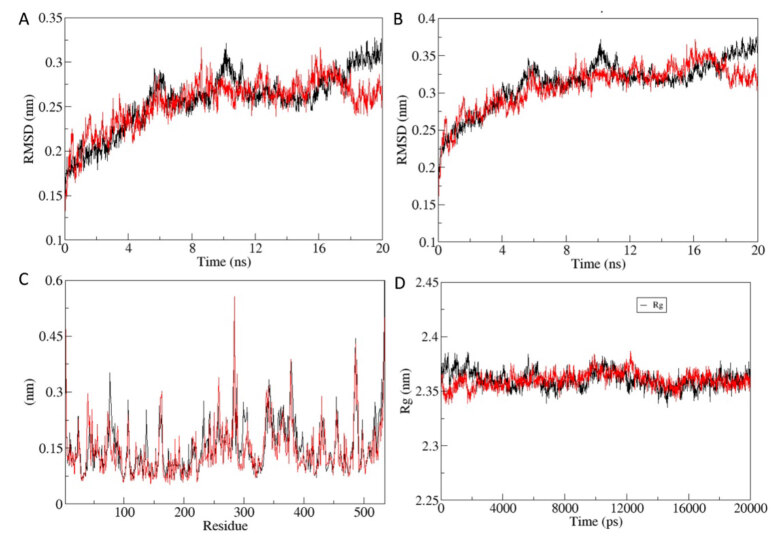
Molecular dynamics (MD) simulation trajectory analysis. A. Backbone root mean square deviation (RMSD) of protein-active molecule complex (Black) and protein-designed molecule complex (Red). B. RMSD of the whole protein of protein-active molecule complex (Black) and protein-designed molecule complex (Red). C. Root mean square fluctuation (RMSF) of protein-active molecule complex (Black) and protein-designed molecule complex (Red). D. Radius of gyration (Rg) of protein-active molecule complex (Black) and protein-designed molecule complex (Red).

Root mean-square deviation (RMSD) analysis computes the average distance between the atoms of 1EVE protein during simulation. The analysis provides insights into protein conformation, stability, and equilibrium of the system during simulation [37]. The average backbone RMSD for protein-active molecule complex and protein designed molecule complex were found to be 0.2565 ± 0.0341 nm and 0.2540 ± 0.0280 nm respectively (Figure 9A). Furthermore, RMSD of the protein for protein-active molecule complex and protein designed molecule complex was found to be 0.3129 ± 0.0336 nm and 0.3090 ± 0.0308 nm, respectively (Figure 9B). RMSD analysis of both complexes indicates that protein designed molecule complex was slightly more stable during the 20 ns MD simulation.

Next, we analyzed the effect of active molecule and designed molecule binding on internal dynamics of target protein by calculating the root mean square fluctuation (RMSF) (Figure 9C). Average RMSF value for protein-active molecule complex and protein designed molecule complex was found to be 0.1531 ± 0.0731 nm and 0.1431 ± 0.0694 nm, respectively. RMSF analysis indicated that binding of designed molecule to the target protein resulted in less fluctuation in comparison to the binding of active molecule. Radius of gyration (Rg) is a parameter to assess the folding of regular secondary structures into 3-dimensional protein structure. Rg indicates change in protein structure compactness and its overall dimension. The effect of active molecule and designed molecule binding on Rg value of the protein was computed (Figure 9D). Average Rg values for protein-active molecule complex and protein designed molecule complex were 2.3604 ± 0.0081 nm and 2.3600 ± 0.0072 nm, respectively. Rg analysis shows that there is no significant difference in compactness of folding of target protein after binding of both active molecule and designed molecule.

**Figure 10:**
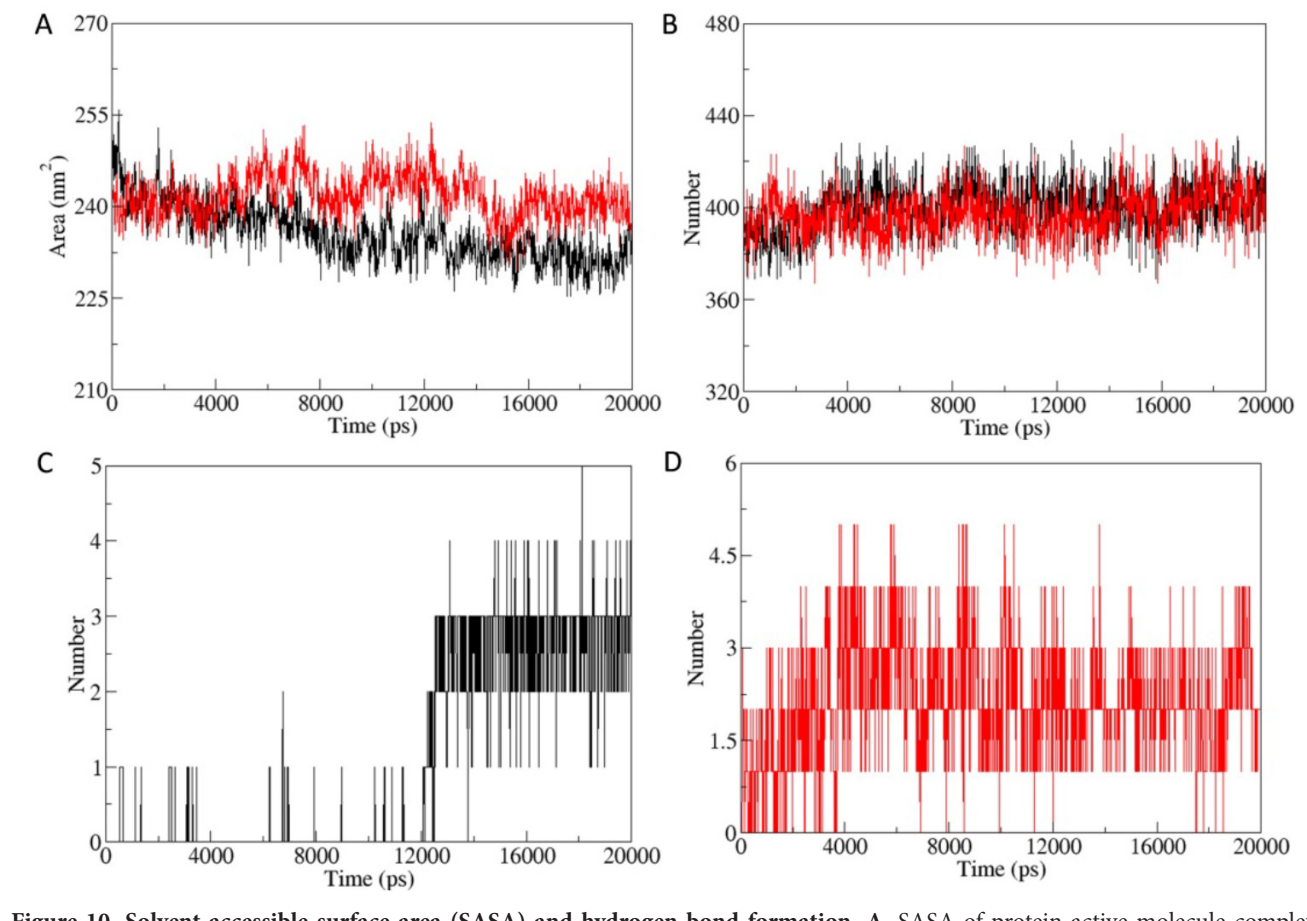
Solvent accessible surface area (SASA) and hydrogen bond formation. A. SASA of protein-active molecule complex (Black) and protein-designed molecule complex (Red). B. Intra-protein hydrogen bond formation in protein-active molecule complex (Black) and protein-designed molecule complex (Red). C. Hydrogen bond formation between protein and ligand in protein-active molecule complex. D. Hydrogen bond formation between protein and ligand in protein-designed molecule complex.

Solvent accessible surface area (SASA) determines the bimolecular surface area assessable to surrounding solvent molecules. The change in SASA for protein-active molecule complex and protein designed molecule complex were analyzed (Figure 10A). Average values of SASA for protein-active molecule complex and protein designed molecule complex were reported as 235.5676 ± 4.5732 nm^2^ and 241.7229 ± 3.5879 nm^2^,respectively. Protein designed molecule complex has significantly high SASA in comparison to protein-active molecule complex.

**Figure 11:**
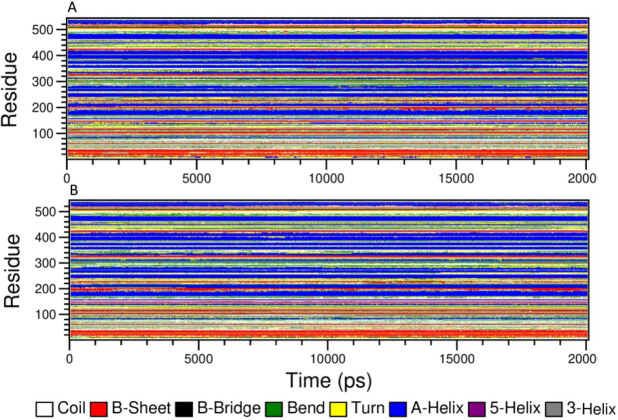
Secondary structure analysis. A. Secondary structure analysis of protein-active molecule complex. B. Secondary structure analysis of proteindesigned molecule complex.

Hydrogen bond formation plays an important role in the stabilization of protein and protein–ligand complex structures by minimizing the energy of the system. Intra-protein (Figure 10B) and protein-ligand hydrogen bonding pattern were studied in both complexes (Figure 10C, 10D). Average value of intra-protein hydrogen bonds in protein-active molecule complex and protein designed molecule complex were 399.9980 ± 10.4525 and 397.3213 ± 9.7869, respectively. Furthermore, hydrogen bond formation between target protein-active molecule (Figure 10C) and target protein-designed molecule (Figure 10D) were also studied. Designed molecule forms significantly high number of hydrogen bonds with target protein in comparison to the active molecule, which indicates the strong binding activity of designed molecule. Further, we conducted secondary analysis for the both complexes to find out the changes in the secondary structures induced by the binding of both active molecule and designed molecule (Figure 11A, 11B). There were no significant changes in secondary structure observed upon binding of both molecules.

## 4. Conclusion 

The outcome of this study gave insights into the design of novel AChE inhibitors for the treatment of Alzheimer. In this paper, a series of 1,2,3-triazole based derivatives previously identified as acetylcholinesterase inhibitors was studied using in silico techniques, such as 3D-QSAR. The overall contour plots analyses revealed that electrostatic and hydrophobic substitutions were found to be critical for increasing the AChE inhibitory activity. Overall, these ﬁndings were very useful for designing six novel AChE inhibitors, among which compound A1 with the highest predictive activity was selected for detailed analyses and compared to the most active compound. Furthermore, validation of 3D-QSAR study was performed by docking-assisted MD simulation study. The comparison illustrated that designed molecule combined with AChE was more stable than the most active compound with the same targeted protein. The designed molecule had a stronger electrostatic and hydrophobic interactions with receptor. The identified structure features for AChE inhibition through docking and MD simulation studies showed a satisfactory correlation with the 3D-QSAR study.
